# In-Situ SEM Observation on Fracture Behavior of Titanium Alloys with Different Slow-Diffusing β Stabilizing Elements

**DOI:** 10.3390/ma13081848

**Published:** 2020-04-15

**Authors:** Wenjing Zhang, Haofeng Xie, Songxiao Hui, Wenjun Ye, Yang Yu, Xiaoyun Song, Lijun Peng, Guojie Huang, Zhen Yang

**Affiliations:** State Key Laboratory of Nonferrous Metals & Processes, GRIMAT Engineering Institute Co., Ltd., Yanqi Economic Development Zone No. 11 XinKe East Street, Beijing 101400, China

**Keywords:** in-situ SEM observation, slow-diffusing β stabilizing elements, 650 °C tensile properties, fracture behavior, interface segregation

## Abstract

The fracture-behaviors of two Ti-Al-Sn-Zr-Mo-Nb-W-Si alloys with different slow-diffusing β stabilizing elements (Mo, W) were investigated through in-situ tensile testing at 650 °C via scanning electron microscopy. These alloys have two phases: the α phase with hcp-structure (a = 0.295 nm, c = 0.468 nm) and the β phase with bcc-structure (a = 0.332 nm). Three-dimensional atom probe (3DAP) results show that Mo and W mainly dissolve in the β phase, and they tend to cluster near the α/β phase boundary. Adding more slow-diffusing β stabilizing elements can improve the ultimate tensile strength and elongation of the alloy at 650 °C. During tensile deformation at 650 °C, microvoids mainly initiate at α/β interfaces. With increases in the contents of Mo and W, the β phase content increases and the average phase size decreases, which together have excellent accommodative deformation capability and will inhibit the microvoids’ nucleation along the interface. In addition, the segregation of Mo and W near the α/β interface can reduce the diffusion coefficient of the interface and inhibit the growth of microvoids along the interface, which are both helpful to improve the ultimate tensile strength and plasticity.

## 1. Introduction

Titanium alloys with exceptional properties of specific strength, creep strength, thermal resistance, corrosion resistance and workability have been developed for high-temperature applications up to 600 °C, such as in compressor disks and the blades of gas turbines in advanced jet engines [1,2]. With increases in the service temperature, the Al equivalent ([Al]eq) of common high-temperature titanium alloys, such as Ti-1100 [3], IMI834 [4] and Ti600 [5], reach the critical value, and the contents of the α stabilizing element and the neutral element have no room to adjust. When designing the composition of high-temperature titanium alloy used at 600 °C, the focus should be on adjusting the contents of the β stabilizing elements. The diffusion rates in the β phase are about two orders of magnitude faster than those in the α phase. Therefore, the volume fraction of the β phase is reduced in high temperature titanium alloys as compared to Ti-6Al-4V, and the β stabilizing element V should be replaced by other slower-diffusing β stabilizing elements. Mo and W are common slow-diffusing β stabilizing elements added in high-temperature titanium alloy, which are characterized by high melting point, high strength, small self-diffusion coefficient, large heat conduction rate and small thermal expansion coefficient. These elements have similar atomic radius and electronegativity to Ti. They have a bcc-structure, which has a high solubility in β-Ti. The addition of these elements can effectively strengthen the β phase and improve the high-temperature strength of titanium alloys [6,7,8,9]. At present, there are few studies on the effects of slow-diffusing β stabilizing elements on the microstructure, mechanical properties and deformation behaviors of high-temperature titanium alloys. Direct reports on observations of the microprocess of fracture at high temperatures are rare. Further studies are required to clarify the micro-mechanism of fracture in titanium alloys at high temperatures, especially the initiation and propagation of microvoids and their interaction with the microstructure. The application of in-situ scanning electron microscopy (SEM) to study the surface morphology evolution of materials during tensile processes greatly facilitates the fracture mechanism analysis [10,11].

In general, microstructure plays an important role in the mechanical properties of titanium alloys, such as strength, ductility, creep resistance and fracture toughness [12,13]. Usually, the bimodal microstructure of the primary α phase (α_p_) and the transformed β phase (β_t_) typically exhibit good room-temperature ductility and high-temperature strength. Therefore, high-temperature titanium alloys prefer this microstructure. Interfaces such as phase boundaries and grain boundaries are ubiquitous defects in titanium alloys, which govern a range of properties such as tensile strength, creep resistance, fatigue resistance and brittleness [14]. Especially at high temperature, interfaces can act as the initiation source or expansion channel of the cracks, which will affect the high-temperature deformation behavior [15]. Such interfaces can either weaken (inter-crystalline fracture, stress corrosion cracking) or strengthen (Hall–Petch effect) polycrystalline metallic materials. The element distribution around the interface can enhance interface cohesion (interface strengthening) or reduce cohesion and bonding strength (interface weakening). As we know, the addition of slow-diffusing β stabilizing elements can enhance the high-temperature strength, but the high-temperature strengthening effects of these elements on an interface have not been studied.

For this paper, novel Ti-Al-Sn-Zr-Mo-Nb-W-Si high-temperature titanium alloys containing different contents of slow-diffusing β stabilizing elements were designed and prepared. The distribution trend of slow-diffusing β stabilizing elements along an interface was revealed via three-dimensional atom probe (3DAP) quantitative analysis. Meanwhile, the effects of slow-diffusing β stabilizing elements on the fracture behaviors (microvoid initiation and propagation) of the alloys were investigated through in-situ SEM.

## 2. Materials and Experimental

The Ti-Al-Sn-Zr-Mo-Nb-W-Si alloy ingots with different contents of slow-diffusing β stabilizing elements were prepared by triple vacuum arc re-melting processing. The ingots were homogenized in β phase region, and then hot forged at 900 °C into bars with a diameter of 12 mm, followed by air cooling. The compositions of the alloys are listed in Table 1. The alloy with high contents of β stabilizing elements was referred to as HBA alloy, and the alloy with low contents of β stabilizing elements as LBA alloy. The β-transus temperature (T_β_) of the alloys was determined experimentally using the phase disappearing method. In this method, they were heat-treated to various temperatures from 900 °C to 1050 °C with minimum temperature of 10 °C, and held for 0.5 h followed by water quenching. The β-transus temperatures (T_β_) of the LBA and HBA alloys were measured to be 1010–1020 °C and 960–970 °C, respectively. In terms of titanium alloys, it is known that the bimodal structure having 20%–30% of equiaxed primary (α_p_) and lamellar transformed β (β_t_) exhibits good ductility and high strength [16]. As a result, the LBA and HBA alloys were heat-treated at a temperature of T_β_-20 °C for 2 h, followed by air cooling, to attain a bimodal structure.

Microstructural observation was performed using an optical microscope (OM: Zeiss Axiovert 200 MAT, ZEISS Group, Oberkochen, Germany) and a scanning electron microscope (SEM: JSM-7001F, JEOL Ltd., Tokyo, Japan). The specimens for microstructural observation were mechanically polished and etched in the Kroll’s reagent (5% HF + 10% HNO_3_ + 85% H_2_O). The 3DAP technique was used to map the element distribution in phase interfaces. The specimens for 3DAP testing were prepared as ultra-sharp needles (tips), with a tip radius of about 20 nm, using a dual-beam focused ion beam (FIB: Helios Nanolab 600i, FEI, Hillsboro, OR, USA). The 3DAP experiments were carried out in a LEAP 5000XR microscope (CAMECA, Paris, France) with a sample temperature of 30 K, a pulse rate of 200 kHz and a detection rate of 0.5%.

The samples for high-temperature tests had a gauge diameter of 5 mm and a gauge length of 25 mm. The tensile tests were performed at 650 °C using an Instron 5582 testing machine (InstronCo., Norwood, MA, USA) equipped with a heating furnace. The strain rate of 0.3/s was used. Three samples of each condition were tested for an average value of ultimate tensile strength (UTS) and elongation (EL).

**Figure 1:**
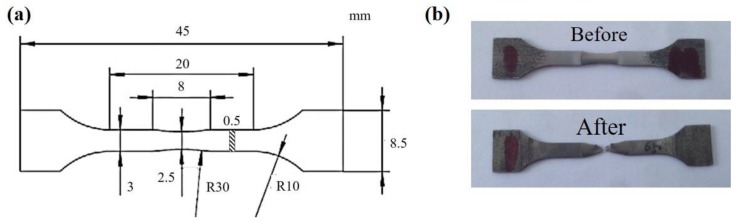
(**a**) Schematic image of in-situ tensile specimens; (**b**) Specimens before and after deformation.

In-situ tensile specimens were machined from the bars via electric discharge machining, and dimensions of the specimens are presented in Figure 1a [15]. The specimens were mechanically ground and then electro-polished in a solution of 6 vol% perchloric acid and 94 vol% acetic acid. In-situ observations were performed in a CS-3400 type SEM (Hitachi, Tokyo, Japan). Tensile tests were performed using a loading stage (maximum load capacity: 1 KN) inside the SEM chamber. The specimen was strained at a strain rate of 0.5 μm/s. The specimens before and after in-situ tensile deformation are shown in Figure 1b.

## 3. Results

### 3.1. Initial Microstructures

**Figure 2:**
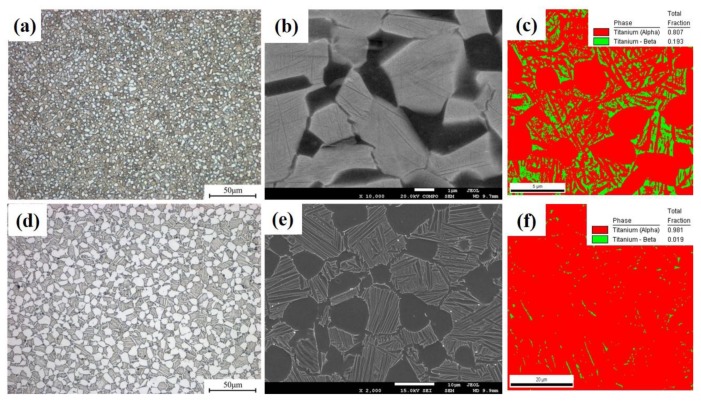
Microstructures of the investigated alloys after heat treatment: (**a**–**c**) HBA; (**d**–**f**) LBA.

Figure 2 shows the microstructures of the alloy samples. Both the samples exhibited a bimodal structure, which consists of equiaxed α_p_ and lamellar β_t_. The effect of grain size refinement as related to traces of slow-diffusing β stabilizing elements can be deduced from Figure 2 and Table 2. As β stabilizers, the addition of Mo and W can affect the precipitation process of the secondary α phase (α_s_) from the β phase. The precipitation of the α phase in the β phase is accompanied by element-diffusion [17]. The diffusion rates of Mo and W in the β phase are much slower than the self-diffusion of β-Ti [18]. The addition of Mo and W could thus enhance the stability of the β phase, and hinder the phase-transformation of the α phase from the β phase, reducing the size of the lamellar α_s_. As shown in Figure 2c,f, the HBA specimen had 19.3% β phase (marked by green), which is almost 10 times that of the LBA specimen. Therefore, it can be concluded that the greater the content of slow-diffusing β stabilizing elements, the finer the grain size and the greater the content of β phase.

### 3.2. In-Situ Observation

**Figure 3:**
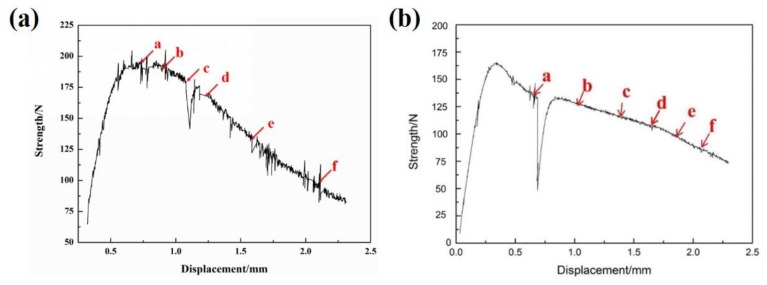
In-situ tensile stress-displacement curve of the investigated alloys during 650 °C tensile process: (**a**) HBA, (**b**) LBA.

**Figure 4:**
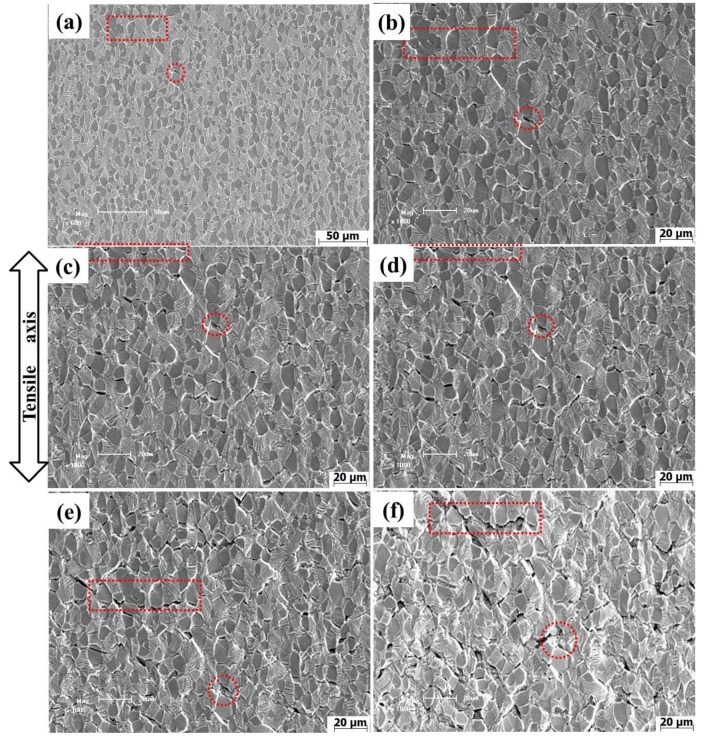
SEM images of the LBA alloy during in-situ tensile process at 650 °C: (**a**) 0.7 mm, (**b**) 1.0 mm, (**c**) 1.4 mm, (**d**) 1.6 mm, (**e**) 1.75 mm, (**f**) 2.0 mm.

**Figure 5:**
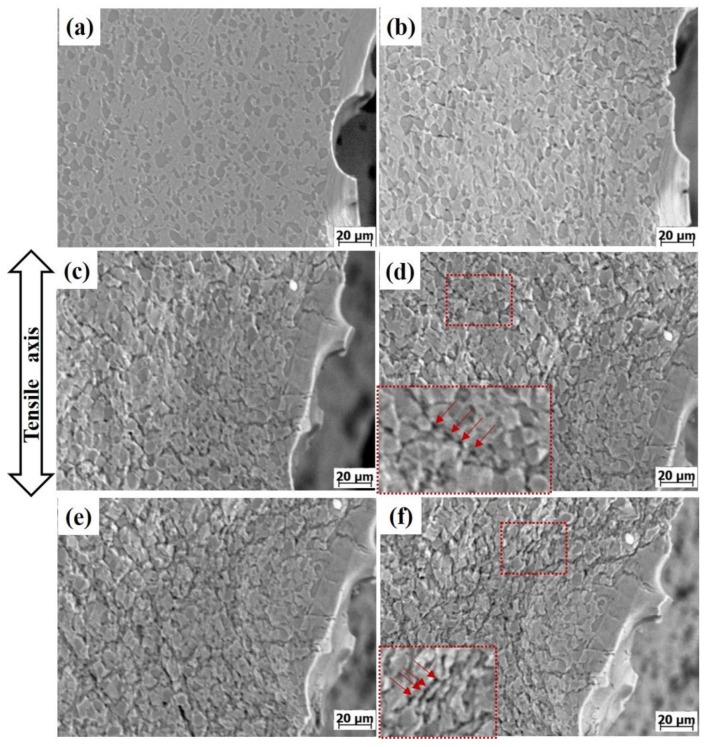
SEM images of HBA alloy during in-situ tensile process at 650 °C: (**a**) 0.75 mm, (**b**) 0.9 mm, (**c**) 1.1 mm, (**d**) 1.25 mm, (**e**) 1.6 mm, (**f**) 2.1 mm.

In-situ SEM tensile deformation was adopted to study the deformation and fracture behavior of the present alloys with different slow-diffusing β stabilizing elements, focusing on the microvoid nucleation and propagation process. Figure 3, Figure 4 and Figure 5 show the typical tensile-displacement curves and in-situ SEM images respectively, taken at sequential load levels during 650 °C tensile processes. Each SEM image corresponds to a point on the curve in Figure 3. During the tensile process, in order to collect SEM images, the tensile process was paused and the load was held, which causes stress relaxation. As shown in Figure 3, slight stress relaxation occurs during the pauses. After imaging, it was loaded again.

Figure 4 shows the in-situ SEM images of the LBA sample during the 650 °C tensile process. It was observed that with the strain increasing, the number of microvoids increases gradually, the distribution of microvoids becomes wider and wider, and the microvoids grow into spindle cracks. The microvoids propagate along the interface and become cracks, as shown in Figure 4b,c. When the deformation is about 1.6 mm, the cracks between the neighboring grains connect together, exhibiting the characteristics of intergranular fracture, as shown in Figure 4d,e. The red rectangular region shows the nucleation and growth process of microvoids in the same location: at point a, there are no microvoids in the rectangular region. At point b, microvoids begin to nucleate at the α_p_/β_t_ phase interface. When the deformation reaches point c, the microvoids grow along the direction of the interface and form cracks. The adjacent cracks connect and form an intergranular crack when the deformation reaches point d. It can be found that during the 650 °C tensile process, the interfacial strength of LBA is low, the microvoids are easy to nucleate at the interface, and the number of microvoids increases rapidly.

Figure 5 shows the in-situ SEM images of the HBA alloy taken at sequential load levels during the 650 °C tensile process. Before the stress reaches UTS, no defect can be observed on the surface of the specimen (Figure 5a). As the strain increases further, tensile stress decreases, and microvoid nucleation along α_p_/β_t_ interfaces is observed when the deformation is about 0.9 mm (Figure 5b), the number of microvoids increasing slowly with increasing the strain. The red rectangular fields in Figure 5d,f are locally enlarged in the lower left corner. From this enlarged view, it can be clearly observed that the crack is composed of circular holes as shown by the arrows; with the deformation increasing, the holes become interconnected. Compared with the LBA sample, the number of microvoids in the HBA sample is lower and the microvoids grow into circular holes. When the deformation reaches a certain degree, a series of “hole strings” will be formed on the interface as shown in the enlarged red rectangular region. As the deformation continues, the adjacent “hole strings” merge along the interface and develop into wavy cracks.

### 3.3. DAP Analysis

From the in-situ tensile test results, it is found that the microvoids mainly tend to nucleate and grow along the α/β interface. It is known that in the bio-modal microstructure of titanium alloys, deformation begins in the soft β phase rather than the hard α phase [19]. The α/β interface has almost a “Burgers orientation” relationship ((0001)_α_//(101)_β_ and [2$\bar{11}$0]_α_//[$\bar{111}$]_β_) [20]. Only the dislocation which slips along the (101)_β_//[$\bar{111}$]_β_ slip system in the β phase can cross the α/β interface easily [21]. It will be difficult for dislocations to cross the interface when the β and α phase do not meet in the relationship, which will produce large stress-concentrations and produce microvoids at the α/β interfaces. In order to strengthen the α/β interfaces, the 3DAP test can be used to investigate the element distribution near the interface.

**Figure 6:**
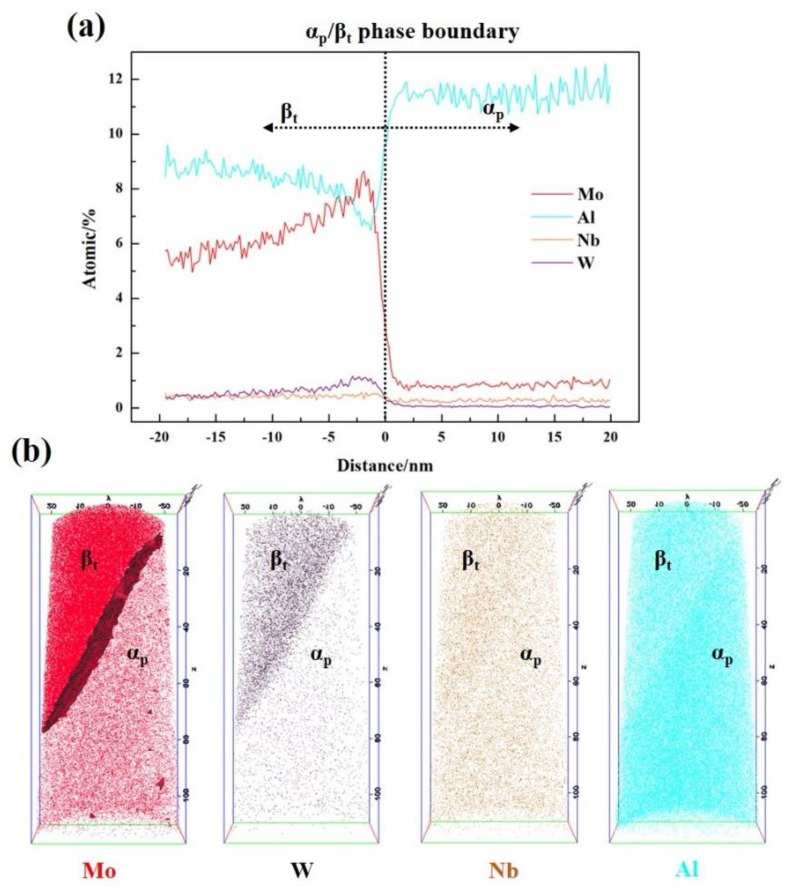
3DAP results from HBA sample: (**a**) The distribution of Mo, W, Nb and Al in β_t_ and α_p_; (**b**) 3DAP maps (40 × 40 × 100 nm) of Mo, W, Nb and Al atoms.

Figure 6 shows the three-dimensional atom distributions of Mo, W, Nb and Al in the HBA specimen. The content of Al in α_p_ is more than that in β_t_. Although Mo, W and Nb are β stabilizing elements, they exhibit different distribution behavior. Mo and W mainly dissolve in the β_t_ structure, and it can be found that they tend to cluster near the α/β phase boundary. The contents of Mo and W are highest at about 2 nm from the α_p_/β_t_ phase boundary in β_t_. However, compared with the β_t_-structure, the content of Nb in the α_p_ phase does decrease significantly.

### 3.4. Mechanical Properties

The tensile properties of the investigated alloys at 650 °C are shown in Table 3. It is observed that the content of slow-diffusing β stabilizing elements has a great influence on the tensile properties. The HBA alloy, having a higher content of β stabilizing elements, had higher strength and ductility (750 MPa, 50.0%) than the LBA alloy (655 MPa, 26.0%). The difference in the tensile properties of the two alloys can be associated with two factors. First, the increment of tensile strength is attributed to the greater amount of slow-diffusing β stabilizing elements, which improves the solid-solution strengthening effect at 650 °C. Second, it is known that the gliding of grain or phase boundaries contributes to the deformation process during high-temperature tensile processes, which reduces the interface strength. The segregation of slow-diffusing β stabilizing elements near the α/β phase boundary can strengthen the interface, which enhances the high-temperature tensile strength.

## 4. Discussion

### 4.1. Effect of Slow-Diffusing β Stabilizing Elements on Nucleation of Microvoids

It is found that the microvoids nucleate at the interfaces during the 650 °C in-situ tensile process. There are two common sites of microvoid nucleation at the interface: (1) microvoid initiates near the triple point of two-dimensional (2d) interface, and (2) interfacial microvoids are produced where intergranular dislocation slip occurs.

#### 4.1.1. Microvoid Initiates near the Triple Point of 2d Interface

**Figure 7:**
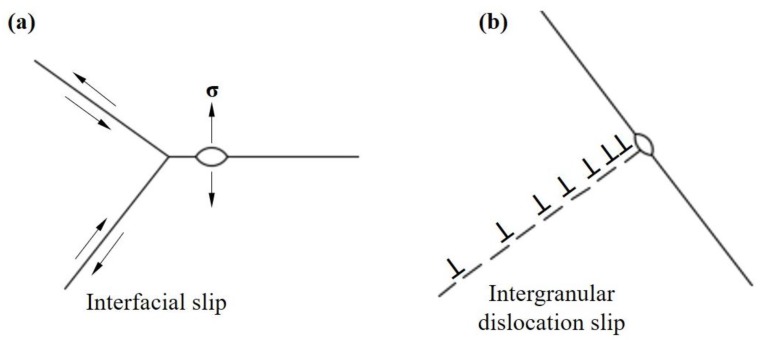
Microvoid nucleation mechanism: (**a**) microvoid initiates near the triple point of 2d interface, (**b**) interfacial microvoid caused by intergranular dislocation slip.

At high temperature, due to the low density of atoms at the interface, the atoms can diffuse easily, and this interfacial slip participates in the deformation process. In order to maintain the continuity of grain boundary, the interfacial slip needs the coordination of the intergranular dislocation slip. Therefore, when the intragranular slip cannot coordinate the interfacial slip, the triple point of the two-dimensional (2d) interface will restrict the interfacial slip, which will cause a large stress-concentration and lead to the formation of microvoids in the interface. Figure 7a is the schematic image of microvoid initiation near the triple point of 2d interface. Riedel [22] proposed the following expression for the stress-distribution at the interface:

(1) $$\sigma \;={0.25\sigma }_{\infty }{\left[\frac{d}{r}\right]}^{1/2}$$

where σ represents the stress-distribution at the interface, *d* represents the grain diameter, σ_∞_ represents the external force and *r* represents the distance from the triple point.

It can be seen that the larger the grain size, the higher the stress, and the easier the formation of microvoids. In this study, the addition of slow-diffusing β stabilizing elements refines the grain size as shown in Figure 2. Compared with the HBA specimen, the LBA specimen had fewer slow-diffusing β stabilizing elements and a larger grain size, therefore it is easier to produce stress-concentration near the triple point at the interface, which leads to the formation of more microvoids during the tensile process.

#### 4.1.2. Interfacial Microvoid Caused by Intergranular Dislocation Slip

Dyson [23] studied the high-temperature deformation behavior of Nimonic80A alloy. He found that the dislocation movement in the grain is concentrated in the slip zone. When the intergranular dislocation slip zone moves to the interface, a large number of dislocations are blocked at the interface, resulting in a large stress-concentration at the interface, which may lead to the nucleation of microvoids. If the adjacent grain has excellent deformation-capability, the stress-concentration can be relieved by the deformation of it. Therefore, the greater the deformation-compatibility of the microstructure, the lower the probability of microvoid nucleation. Figure 7b is the schematic image of interfacial microvoids caused by intergranular dislocation slip. In this study, the addition of slow-diffusing β stabilizing elements refines the grain size and enhances the stability of the β phase as shown in Figure 2. During the 650 °C tensile process, a finer grain and greater β phase had an excellent capacity for deformation, which made it easier to start more slip systems and release the stress-concentration at the interface. With regard to LBA, HBA has more slow-diffusing β stabilizing elements, which results in finer grain and more β phase, making the nucleation of microvoids more difficult during 650 °C tensile process.

It can be concluded that with the slow-diffusing β stabilizing elements increasing, the grain size decreases and the content of β phase increases, which creates difficulties for microvoid nucleation at the interphase during the 650 °C tensile process. This is also the reason why the tensile strength of the HBA specimen, with more slow-diffusing β stabilizing elements, is higher than that of the LBA specimen.

### 4.2. The Effect of Slow-Diffusing β Stabilizing Elements on the Growth of Microvoids

**Figure 8:**
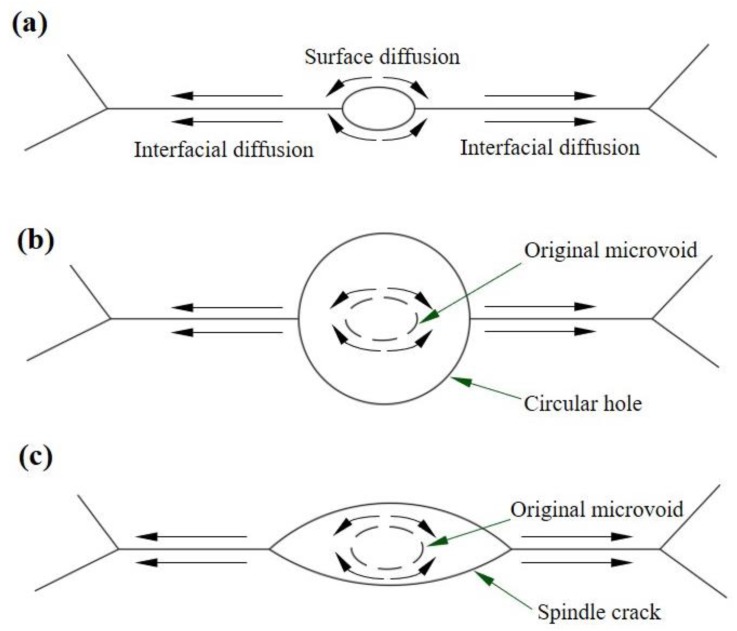
(**a**) The model of microvoid growing with diffusion; (**b**) the model of microvoid growing up into a circularshape cavity; (**c**) the model of microvoid growing up into a spindle shape crack.

In this study, there are two different shapes the microvoids grow into: circular and spindle shape. Hull and Rimmer [24] had proposed that the microvoids grow with diffusion, and the growth of microvoids require vacancies. As shown in Figure 8a, the growth of microvoids is related to interfacial diffusion and surface diffusion, and the shape of the microvoid is controlled by the slowest of these two processes. When the microvoid is nucleated at the interface, the atoms will diffuse from the surface of the microvoid to the interface, and then continue to diffused along the interface. Meanwhile, the microvoid will absorb the vacancies and grow up. When the interface diffusion is slower than the surface diffusion, the growth rate of the microvoid is controlled by the interface diffusion. Just like the HBA alloy studied in this paper, there are more Mo and W added to this alloy, and these two elements tend to cluster near the α/β phase boundary as shown in Figure 6. When the high melting-point elements are present at the interface, the atomic activation energy is increased and the diffusion coefficient of the interface is reduced, which will lead to the difficulty in microvoid propagation along the interface, and cause the microvoid to grow into a circular cavity, as shown in Figure 8b. When the interface diffuses faster than the surface, the growth rate of the micropore is controlled by the surface diffusion, and the micropore expands into a spindle shape, as shown in Figure 8c. Just like the LBA alloy studied in this paper, the addition of Mo and W in the alloy is lesser, which makes the interface diffusion faster than the surface diffusion, and leads to the microvoid growing more quickly along the interface and into a spindle crack during the 650 °C tensile process.

It can be concluded that the segregation of Mo and W near the α/β interface can reduce the diffusion coefficient of the interface and inhibit the growth of microvoids along the interface, which is helpful to improve the tensile plasticity. This is also the reason why the tensile elongation of the HBA specimen is higher than that of the LBA specimen.

## 5. Conclusions

650 °C fracture behavior of Ti-Al-Sn-Zr-Mo-Nb-W-Si alloys with different contents of slow-diffusing β stabilizing elements were evaluated using in-situ tensile SEM surface observations. It was found that:

(1) Microvoids mainly initiate at α/β interfaces, and the investigated alloys present microvoid coalescence intergranular fracture characteristics.
(2) With the contents of Mo and W increasing, the β phase content increases and the grain size decreases, which leads to the difficulty of microvoid nucleation at the interface and enhances the ultimate tensile strength.
(3) The segregation of Mo and W near the α/β interface can reduce the diffusion ability of the interface and inhibit the growth of microvoids along the interface, which is helpful to improve the plasticity. The microvoids grow into circular shapes in the HBA specimen with more slow-diffusing β stabilizing elements, and spindle shapes in the LBA specimen with less slow-diffusing β stabilizing elements.

## Figures and Tables

**Table 1:** Chemical compositions of the alloys (wt.%).

Sample Name	Composition
HBA	Ti-6.5Al-2Sn-4Zr-4Mo-2Nb-1W-0.2Si
LBA	Ti-6.5Al-2Sn-4Zr-1Mo-3Nb-0.5W-0.2Si

**Table 2:** Microstructural parameters of the investigated alloys after heat treatment.

Samples	α_p_/μm	β_t_/μm	α_s_/nm	β Phase Content/%
HBA	2	3–6	100	19.3
LBA	6	13–19	300	1.9

**Table 3:** 650 °C tensile properties of the investigated alloys.

Samples	Tensile Strength/MPa	Elongation/%
HBA	750	50.0
LBA	655	26.0

## References

[1] Narayana P.L., Kim S.W., Hong J.K., Reddy N.S., Yeom J.T. (2018). Tensile properties of a newly developed high-temperature titanium alloy at room temperature and 650 °C. Mater. Sci. Eng. A.

[2] Guo R., Liu B., Xu R.J., Cao Y.K., Qiu J.W., Chen F., Yan Z.Q., Liu Y. (2020). Microstructure and mechanical properties of powder metallurgy high temperature titanium alloy with high Si content. Mater. Sci. Eng. A.

[3] Chandravanshi V.K., Bhattacharjee A., Kamat S.V., Nandy T.K. (2014). Influence of thermomechanical processing and heat treatment on microstructure, tensile properties and fracture toughness of Ti-1100-0.1B alloy. J. Alloy. Compd..

[4] Wang S.Q., Li W.Y., Zhou Y., Li X., Chen D.L. (2016). Tensile and fatigue behavior of electron beam welded dissimilar joints of Ti–6Al–4V and IMI834 titanium alloys. Mater. Sci. Eng. A.

[5] Ding C., Shi Q., Liu X., Zheng L., Li R.X., Huang Z.X., Yu B.Y., Wei W. (2019). Microstructure and mechanical properties of PM Ti600 alloy after hot extrusion and subsequent annealing treatment. Mater. Sci. Eng. A.

[6] Gogia A.K. (2005). High-temperature titanium alloys. Defence. Sci. J..

[7] Zhang W.J., Song X.Y., Hui S.X., Ye W.J. (2017). The effects of Mo content on microstructure and high temperature tensile behavior of Ti-6.5Al-2Sn-4Zr-xMo-2Nb-1W-0.2Si titanium alloys. Mater. High Temp..

[8] Zhang W.J., Song X.Y., Hui S.X., Ye W.J., Wang Y.L., Wang X.X. (2013). Effect of single annealing on the microstructure and mechanical properties of BTi-6431S titanium alloy. Chin. J. Nonferrous Met..

[9] Zhang W.J., Song X.Y., Hui S.X., Ye W.J., Wang W.Q. (2018). Phase precipitation behavior and tensile property of a Ti–Al–Sn–Zr–Mo–Nb–W–Si titanium alloy. Rare Metals..

[10] Yuan Z.Z., Dai Q.X., Cheng X.N., Chen K.M., Pan L., Wang A.D. (2006). In situ SEM tensile test of high-nitrogen austenitic stainless steels. Mater. Charact..

[11] Shao H., Zhao Y.Q., Ge P., Zeng W.D. (2013). In-situ SEM observations of tensile deformation of the lamellar microstructure in TC21 titanium alloy. Mater. Sci. Eng. A.

[12] Omprakash C.M., Statyanarayana D.V.V., Kumar V. (2010). Effect of microstructure on creep and creep crack growth behavior of titanium alloy. Trans. Indian Inst. Met..

[13] Zhang W.J., Song X.Y., Hui S.X., Ye W.J., Wang Y.L., Wang W.Q. (2014). Tensile behavior at 700 ℃ in Ti–Al–Sn–Zr–Mo–Nb–W–Si alloy with a bi-modal microstructure. Mater. Sci. Eng. A.

[14] Raabe D., Herbig M., Sandlobes S., Li Y., Tytko D., Kuzmina M., Ponge D., Choi P.-P. (2014). Grain boundary segregation engineering in metallic alloys: A pathway to the design of interfaces. Curr. Opin. Solid State Mater. Sci..

[15] Zhang W.J., Song X.Y., Hui S.X., Ye W.J. (2017). In-situ SEM observations of fracture behavior of BT25y alloy during tensile process at different temperature. Mater. Design..

[16] Neal D.F., Lütjering G., Zweicker U., Bunk W. (1985). Titanium: Science and Technology.

[17] Wang Z.H., Xia C.Q., Peng X.M., Chen Z.H., Li X.X. (2010). Effect of heat treatment on microstructure and mechanical properties of Ti62421s high temperature titanium alloy. Chin. J. Nonferrous Met..

[18] Lütjering G., Williams J.C. (2007). Titanium.

[19] Kim J.S., Kim J.H., Lee Y.T., Park C.G., Lee C.S. (1999). Microstructural analysis on boundary sliding and its accommodation mode during superplastic deformation of Ti-6Al-4V alloy. Mater. Sci. Eng. A.

[20] Wang X., Jahazi M., Yue S. (2006). Substructure of high temperature compressed titanium alloy IMI 834. Mater. Sci. Eng. A.

[21] Ambard A., Guétaz L., Louchet F., Guichard D. (2001). Role of interphases in the deformation mechanisms of an α/β titanium alloy at 20 K. Mater. Sci. Eng. A.

[22] Riedel H. (1987). Fracture at High Temperature.

[23] Dyson B.F., Rodgers M.J. (1977). Intergranular creep fracture in Nimonic 80A under conditions of constant cavity density. Phys. Metall. Fract..

[24] Hull D., Rimmer D.E. (1959). The growth of grain-boundary voids under stress. Philos. Mag..

